# Age- and Genotype-Specific Effects of the Angiotensin-Converting Enzyme Inhibitor Lisinopril on Mitochondrial and Metabolic Parameters in *Drosophila melanogaster*

**DOI:** 10.3390/ijms19113351

**Published:** 2018-10-26

**Authors:** Karis A. Ederer, Kelly Jin, Sarah Bouslog, Lu Wang, Gregory S. Gorman, Glenn C. Rowe, Peter Abadir, Daniel Raftery, Douglas Moellering, Daniel Promislow, Patricia Jumbo-Lucioni, Maria De Luca

**Affiliations:** 1 Departments of Nutrition Science, University of Alabama at Birmingham, Birmingham, AL 35294, USA; karis.antonia.ederer@live.mercer.edu (K.A.E.); sbouslog@uab.edu (S.B.); dmoellering@uab.edu (D.M.); pjumbolu@samford.edu (P.J.-L.); 2 Department of Kinesiology, University of Alabama at Birmingham, Birmingham, AL 35294, USA; 3 Department of Physical Therapy, Mercer University, Atlanta, GA 30341, USA; 4 Department of Pathology, University of Washington, Seattle, WA 98195, USA; kellyjin@uw.edu (K.J.); promislo@u.washington.edu (D.P.); 5 Department of Biology, University of Alabama at Birmingham, Birmingham, AL 35233, USA; 6 Department of Environmental and Occupational Health Sciences, University of Washington, Seattle, WA 98195, USA; lwang3@uw.edu; 7 Department of Pharmaceutical, Social and Administrative Sciences, Samford University, Birmingham, AL 35229, USA; ggorman@samford.edu; 8 Pharmaceutical Sciences Research Institute, Samford University, Birmingham, AL 35229, USA; 9 Division of Cardiovascular Disease, University of Alabama at Birmingham, Birmingham, AL 35233, USA; glennrowe@uabmc.edu; 10 Division of Geriatric Medicine and Gerontology, Johns Hopkins School of Medicine, Baltimore, MD 21234, USA; pabadir1@jhmi.edu; 11 Department of Anesthesiology & Pain Medicine, University of Washington, Seattle, WA 98195, USA; draftery@uw.edu; 12 Department of Biology, University of Washington, Seattle, WA 98195, USA

**Keywords:** aging, angiotensin-converting enzyme inhibitors, nutrient metabolism, genetic background, nutritional stress

## Abstract

The angiotensin-converting enzyme (ACE) is a peptidase that is involved in the synthesis of Angiotensin II, the bioactive component of the renin-angiotensin system. A growing body of literature argues for a beneficial impact of ACE inhibitors (ACEi) on age-associated metabolic disorders, mediated by cellular changes in reactive oxygen species (ROS) that improve mitochondrial function. Yet, our understanding of the relationship between ACEi therapy and metabolic parameters is limited. Here, we used three genetically diverse strains of *Drosophila melanogaster* to show that Lisinopril treatment reduces thoracic ROS levels and mitochondrial respiration in young flies, and increases mitochondrial content in middle-aged flies. Using untargeted metabolomics analysis, we also showed that Lisinopril perturbs the thoracic metabolic network structure by affecting metabolic pathways involved in glycogen degradation, glycolysis, and mevalonate metabolism. The Lisinopril-induced effects on mitochondrial and metabolic parameters, however, are genotype-specific and likely reflect the drug’s impact on nutrient-dependent fitness traits. Accordingly, we found that Lisinopril negatively affects survival under nutrient starvation, an effect that can be blunted by genotype and age in a manner that partially mirrors the drug-induced changes in mitochondrial respiration. In conclusion, our results provide novel and important insights into the role of ACEi in cellular metabolism.

## 1. Introduction

The circulating renin-angiotensin system (RAS) is a hormonal system whose primary function is to regulate arterial pressure as well as water and sodium homeostasis [1]. The main effector of RAS is Angiotensin (Ang) II, which is produced by enzymatic sequential cleavage of peptides derived from the liver-produced angiotensinogen. Angiotensinogen is converted by renin to Ang I, which in turn is converted to Ang II by the action of the angiotensin-converting enzyme (ACE) [1]. Ang II exerts its actions by binding with equal affinity to two main G protein-coupled receptors, type-1 receptor (AT_1_R) and type-2 receptor (AT_2_R), which have different tissue distribution and opposite effects on vascular tone [2]. Within the past 15 years, it has become evident that several RAS components are present in almost every organ (local RAS), where they exert diverse organ-specific physiological and pathophysiological functions through the action of de novo synthesized Ang II. Local RASs operate in concert with the systemic RAS, but also independently [3,4]. 

Two drug classes that inhibit RAS by directly targeting Ang II, the ACE inhibitors (ACEi) and the angiotensin receptor blockers (ARBs), are widely used in clinical practice to manage cardio-vascular disorders and chronic kidney disease [2]. More recent evidence suggests that administration of ACEi or ARBs can also improve physical function in older individuals with impairment of daily activities [5] and in physically independent elderly people [6]. Moreover, ACEi or ARB-induced blockade of RAS has been shown to reduce the incidence of type-2 diabetes in patients with heart failure or at risk for coronary artery disease [7] and ameliorate skeletal muscle insulin sensitivity in mammalian models [8]. These recent findings highlight the significant effects of these drugs on metabolic parameters and the complexity of the biology of mammalian RAS.

Recently, several investigators have proposed that the beneficial effects of ACEi and ARBs on aging and a wide spectrum of chronic metabolic diseases are partly due to the capacity of these drugs to reduce cellular ROS production and thereby preserve the physiological phosphorylation state of the mitochondria [9,10,11,12]. This idea is particularly intriguing considering the solid evidence that Ang II binding to the AT_1_ receptor stimulates the production of ROS via regulation of nicotinamide adenine dinucleotide phosphate-oxidase (NADPH) oxidase activity [12,13]. Ang II-induced ROS, in turn, oxidize downstream redox-sensitive pathway targets involved in cellular processes, such as cell growth, inflammation, and fibrosis that promote tissue remodeling and repair [12]. Additionally, clinical evidence indicates that the renal and cardiac benefits of ACEi and ARBs in patients with hypertension and cardiovascular disease are somewhat independent of their blood pressure-lowering effects [3,4]. However, disentangling the vascular hemodynamic effects of these drugs from their direct effects on cellular metabolism remains a challenge in humans and in vivo vertebrate models. To tackle this issue, in this study we used the invertebrate model *D. melanogaster*, which is an attractive model to study the relationship between ACEi therapy, metabolism, and aging for several reasons. First, fly orthologues of human ACE, called angiotensin-converting enzyme (AnCE) and angiotensin-converting enzyme related (ACER), have been well described [14,15] and, like human ACE, regulate heart function [16]. Second, the activity of AnCE is inhibited by the same drugs (including Lisinopril) that inhibit human ACE through a similar mechanism [17]. Third, mitochondrial morphology in *Drosophila* indirect flight muscles (found in the insect thorax) has been shown to be a sensitive pharmacological target of the ARB Losartan [18], suggesting a potential relationship between RAS-like components and muscle mitochondrial-related phenotypes in *Drosophila*.

Previously, we used wild-derived inbred strains of the *Drosophila* Genetic Reference Panel (DGRP) to show that there is significant within-population genetic variability for mitochondrial function in the thoraces of young flies [19]. Here, we fed newly eclosed male flies from three distinct DGRP strains (DGRP_73, DGRP_229, and DGRP_304) with food containing either 1 mM Lisinopril or no drug for one week or three weeks. The objective of the study was to investigate whether Lisinopril treatment affects thoracic hydrogen peroxide (H_2_O_2_) levels, mitochondrial function and content, and metabolomic profiles and if its effects are influenced by genetic factors and/or age. The three DGRP strains were chosen because of their genetically-based differences in average starvation resistance [20], an essential fitness trait that is influenced by alterations in muscle substrate metabolism [21,22]. We previously showed a positive correlation between thoracic mitochondrial respiration and starvation resistance in the DGRP strains [19]; therefore, we reasoned that, if present, genotype-specific effects of Lisinopril on mitochondrial and metabolic parameters could be mediated by the same genetic factors that affect the capacity of the fly to survive under nutrient starvation.

We report that Lisinopril administration affects thoracic mitochondrial function, mitochondrial content, and H_2_O_2_ levels as well as starvation survival in *D. melanogaster*, strongly suggesting the existence of evolutionarily conserved physiological mechanisms linking ACEi and cellular energy metabolism. We also reveal metabolic pathways perturbed by Lisinopril treatment. Furthermore, we determine that Lisinopril effects on *Drosophila* mitochondrial and metabolic parameters are strongly influenced by genetic background and advancing aging, which therefore should be considered when AnCE/ACEi studies are designed.

## 2. Results

### 2.1. Lisinopril Treatment Alters Thoracic Mitochondrial Function and Content as well as H_2_O_2_ Levels in a Genotype- and Age-Specific Manner

**Figure 1:**
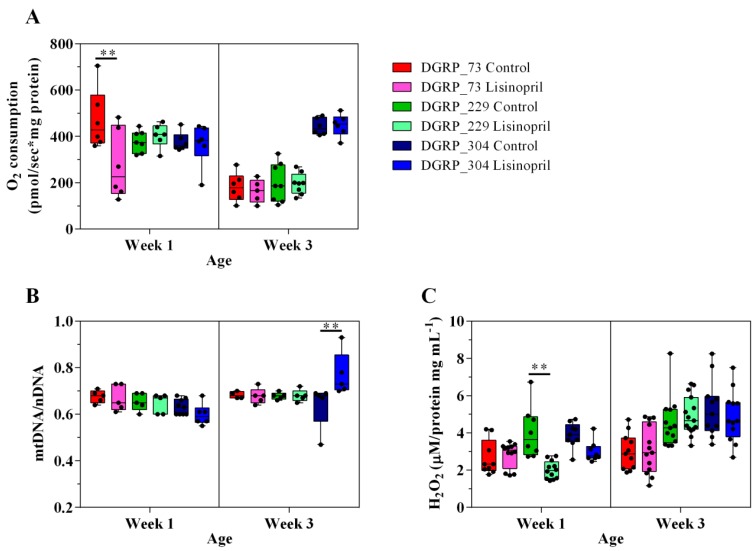
Lisinopril treatment alters thoracic mitochondrial function and content as well as H_2_O_2_ levels in a genotype- and age-specific manner. (**A**–**C**) Lisinopril significantly reduces state 3 respiration of mitochondria isolated from the thoraces of DGRP_73 young flies (panel **A**), increases thoracic mitochondrial content in DGRP_304 middle-aged flies (panel **B**), and decreases H_2_O_2_ levels in DGRP_229 young flies (panel **C**). Box and whiskers plots denote individual data points separated by a line representing the group median. Each individual value is plotted as a dot superimposed on the boxplots. In all panels, ** *p* < 0.01, obtained from Tukey post hoc tests for multiple comparisons.

In this study, we used the NAD^+^-linked substrates pyruvate/proline to measure the oxygen consumption rate in the mitochondria isolated from the thoraces of one-week and three-week-old DGRP flies. State 3 respiration refers to the oxygen consumed by isolated mitochondria in the presence of saturating amounts of respiratory substrate and ADP and is an index of oxidative phosphorylation (OxPhos) capacity. We observed a significant effect of genotype and age on thoracic mitochondrial OxPhos capacity (see Supplementary Table S1). However, the effect of age is not homogenous across the three genotypes. Indeed, while mitochondria isolated from the thoraces of three-week-old DGRP_73 and DGRP_229 flies had a significantly lower OxPhos capacity (56%, *p* < 0.0001 and 49%, *p* < 0.0001, respectively) than those from younger flies, no age-related decline was observed in DGRP_304 (Figure 1A). This latter finding is very exciting because it corroborates previous work in *D. melanogaster* [23] and humans [24] showing a gradual decline in skeletal muscle mitochondrial function with aging and it also suggests that genetic factors influence this decline. Furthermore, we found that Lisinopril significantly reduces mitochondrial state 3 respiration but it does so in a genotype- and age-dependent manner (Figure 1A and Supplementary Table S1). Unlike mitochondria isolated from DGRP_229 and DGRP_304 flies fed Lisinopril, those isolated from DGRP_73 flies consumed approximately 41% less oxygen during state 3 respiration than untreated flies but only at the younger age (Figure 1A). 

It is well established that mitochondrial coupling can be reduced by a basal leak of protons across the mitochondrial inner membrane [25]. Given that basal proton leak is greatest under non-phosphorylating conditions (i.e., oxygen is consumed in the presence of respiratory substrate and absence of ADP) in isolated mitochondria [25], we assessed the mitochondrial basal state or state 2 and oligomycin-induced state 4 (state 4o) respiration in the three *Drosophila* strains. We found not only a significant effect of genotype on both mitochondrial traits but also that the age-dependent decrease in mitochondrial state 2 and state 4o was not present in all the strains (Supplementary Table S1). However, there was no significant effect of Lisinopril on state 2 or state 4o respiration rates (Supplementary Table S1). 

To corroborate that the effect of Lisinopril on mitochondrial state 3 respiration is independent of mitochondrial content, we measured the mitochondrial DNA (mtDNA)/nuclear DNA (nDNA) ratio in the thoraces of the three *Drosophila* strains. In addition, given the role played by mammalian Ang II in NADPH-induced ROS production [13], we quantified thoracic H_2_O_2_ levels. Similar to mitochondrial respiration, there were significant Lisinopril-by-genotype-by-age interaction effects on both thoracic mitochondrial content and H_2_O_2_ levels (Figure 1B,C, respectively, and Supplementary Table S2). DGRP_304 flies fed with Lisinopril displayed higher (17%) mtDNA/nDNA levels than DGRP_304 untreated flies, but only at three weeks of age (Figure 1B). On the other hand, Lisinopril significantly reduced (50%) thoracic H_2_O_2_ levels only in DGRP_229 younger flies (Figure 1C).

Taken together, these results suggest that Lisinopril alters mitochondrial OxPhos capacity and content as well as ROS production in *D. melanogaster*, but does so through different mechanisms that are influenced by genetic background and age.

### 2.2. A Thoracic Metabolomic Signature Is Associated with Lisinopril Treatment

**Figure 2:**
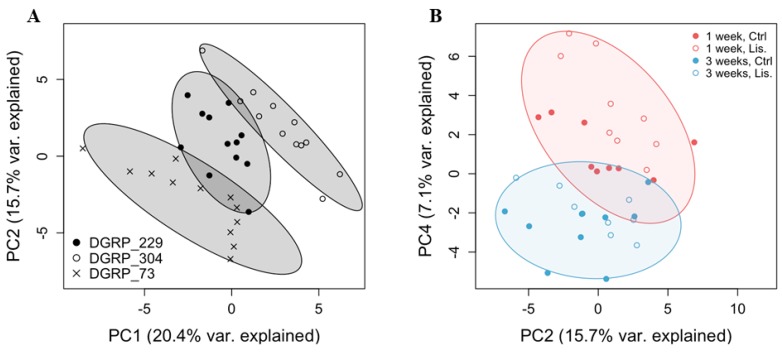
Principal component analysis of thoracic metabolomic profiles. (**A**,**B**) Principal component (PC) scores are produced by metabolic features detected by LC/MS in both positive and negative ion modes. While PC1 and PC2 separate samples by genotype (panel **A**), PC4 almost completely separates samples by age (panel **B**), and it is plotted here against PC2. In both panels, ellipses represent 90% confidence intervals of the groups.

**Figure 3:**
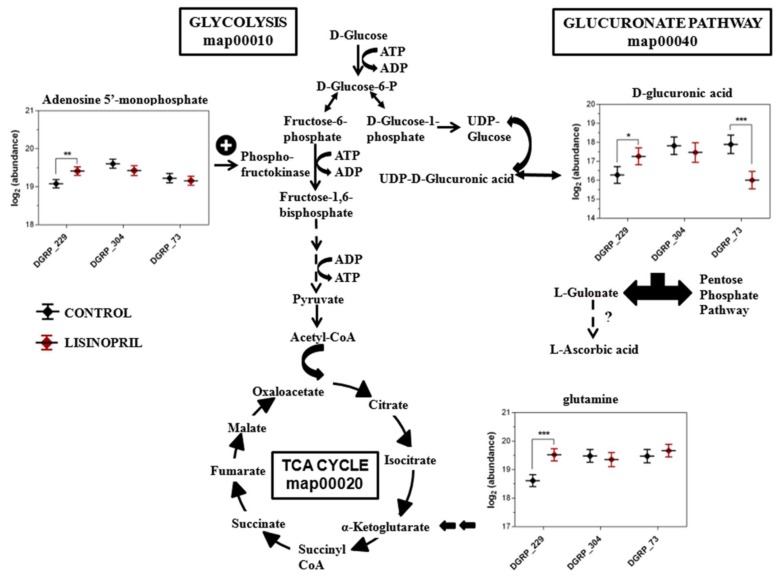
Three metabolites with genotype-specific changes in response to Lisinopril are involved in glycolysis regulation, the glucoronate pathway, and the tricarboxylic acid (TCA) cycle. Data reported on the plots represent the mean log^2^ abundance of three replicate samples for each treatment and genotype group. * *p* < 0.05, ** *p* < 0.05, *** *p* < 0.001 after Benjamini and Hochberg’s adjustment for multiple comparisons. Error bars represent the 95% confidence interval. ?: Hypothetical connection in *Drosophila melanogaster*.

**Figure 4:**
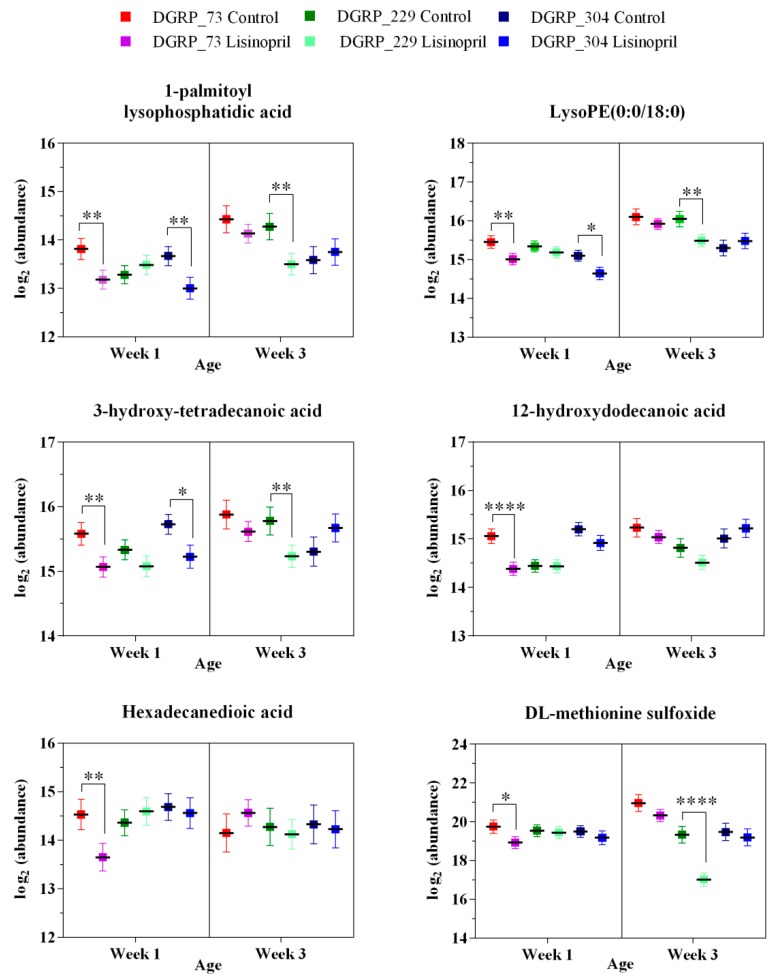
Thoracic metabolites with genotype- and age-specific changes in response to Lisinopril. Data reported on the plots denote the mean log^2^ abundance of three replicate samples for each treatment, genotype, and age group. * *p* < 0.05, ** *p* < 0.01, and **** *p* < 0.0001 after Benjamini and Hochberg’s adjustment for multiple comparisons. Error bars represent the 95% confidence interval.

Muscle is a highly plastic tissue. Pathophysiological and environmental perturbations lead to alterations in mitochondria bioenergetics and energy substrates in the muscle of diverse species, including *D. melanogaster* [21]. In this light, we next sought to investigate whether the effect of Lisinopril on thoracic mitochondrial function and content was accompanied by changes in substrate metabolism. To do this, we used untargeted high-resolution metabolomics and detected 2674 and 1231 metabolite features in positive ionization mode and negative ionization mode, respectively (Supplementary Table S3). After data pretreatment and filtering, the total metabolite features resulted in 2096 features in positive ionization mode and 916 features in negative ionization mode. To identify potential Lisinopril effects on the metabolomic profiles, we first performed an unsupervised PCA on pooled metabolite features. We found that PC1 and PC2, which together capture the greatest variance across the dataset (36%), clearly separated the samples by genotype (Figure 2A). PC4 alone, which captures 7% of the metabolome variance, almost entirely separated samples according to age (Figure 2B). Furthermore, although there was no obvious separation of samples by treatment across the first six PCs, PC4 qualitatively seemed to separate one-week-old samples by Lisinopril treatment, with week one Lisinopril samples appearing to have more “youthful” PC4 scores compared to their age-matched control-treated counterparts (Figure 2B). As such, we next ran univariate analyses to identify changes in individual feature metabolites associated with genotype, age, and treatment. We were also interested in depicting individual metabolites showing changes in response to Lisinopril that are (i) dependent only on genotype (Lisinopril-by-genotype interaction controlling for age effect); (ii) dependent only on age (Lisinopril-by-age interaction controlling for genotype effect); and (iii) dependent on genotype and age (Lisinopril-by-genotype-by-age interaction effects). We found 1912 and 651 features that significantly (FDR < 0.1) vary across the three strains and between ages, respectively, as well as 313 features that showed significant changes in their levels after Lisinopril treatment (Supplementary Table S4A–C, respectively). Additionally, we detected 19, 1 and 37 metabolite features with levels that vary significantly in response to Lisinopril in a genotype-specific, age-specific, or genotype- and age-specific manner, respectively (Supplementary Table S4D–F, respectively). Of the metabolites that showed genotype-specific changes in response to Lisinopril, three, adenosine 5′-monophosphate (AMP), d-glucuronic acid, and glutamine, are involved in glycolysis regulation, the glucuronate pathway, and the tricarboxylic acid cycle, respectively (Figure 3). While the Lisinopril treatment significantly increased the abundance of AMP (26%), d-glucuronic acid (97%), and glutamine (87%) in DGRP_229 flies, it significantly reduced d-glucuronic acid abundance (73%) in DGRP_73 flies. None of these metabolites appears to be affected by Lisinopril in DGRP_304 flies (Figure 3). Metabolites perturbed by the drug in a genotype- and age-specific manner include 1-palmitoyl lysophosphatidic acid, hexadecanedionic acid, and DL-methionine sulfoxide (Figure 4 and Supplementary Table S4F). Furthermore, we observed that five of the thoracic metabolites that are affected by the Lisinopril treatment in a genotype-specific or genotype- and age-specific manner are phosphatidylethanolamines (PE) (Supplementary Table S4D,F).

**Figure 5:**
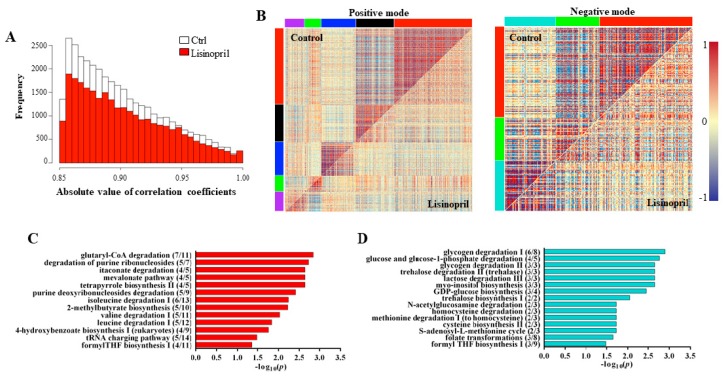
Lisinopril perturbs the thoracic metabolic network structure. (**A**) Distribution of extreme pairwise metabolic feature correlation coefficients. Pairwise Pearson correlations were performed on all metabolic features from positive and negative mode within Control samples (*n* = 18) and Lisinopril samples (*n* = 18). Shown are the distributions of correlation coefficients with an absolute value greater than 0.85 for each treatment. (**B**) Heat maps of correlated metabolite features detected in positive ion mode (three modules) and negative ion mode (five modules). Each point represents the correlation between two metabolite features and the color scale bar indicates the value of the correlations. (**C**,**D**) Representative pathways in the red module for metabolite features detected in positive mode (panel **C**) and in the turquoise module for metabolite features detected in negative mode (panel **D**). Numbers in parentheses indicate overlap size/pathway size.

In an effort to understand not only how single metabolites vary with Lisinopril treatment but also how metabolites co-vary with each other in either treatment, we performed pairwise correlation analysis across all features in control and Lisinopril samples. In comparing the distribution of all possible pairwise correlation coefficients among metabolites, we found that control samples had a much greater proportion of high correlation coefficients (|*r*| > 0.85) than Lisinopril-treated samples (Figure 5A). This result strongly suggests that Lisinopril treatment may cause a loss of regulation across metabolic features. To provide insight into the biological relevance of these metabolite features, we first performed a differential co-expression analysis and identified three and five modules (or clusters) of differentially co-regulated metabolites between Lisinopril-treated and control flies for negative and positive ion mode metabolites, respectively (Figure 5B). We then ran the features from each identified module through the metabolite prediction program *Mummichog* [26] to perform pathway enrichment analysis. Supplementary Table S5 reports the full list of significant pathways in each module. Among the significant pathways, we observed enrichment for pathways related to the mevalonate metabolic pathway and salvage of adenine and hypoxanthine in the red module for metabolites detected in positive mode (Figure 5C). Further, the turquoise module for metabolites detected in negative mode was enriched for pathways related to glycogen degradation, glycolysis, methionine metabolism, and formyl tetrahydrofolate (THF) synthesis (Figure 5D). Additional pathway modules were related to TAG biosynthesis and the de novo biosynthesis of NAD from the amino acid tryptophan (see Supplementary Table S5). 

### 2.3. Lisinopril Negatively Impacts Survival under Nutrient Starvation but the Effect Can Be Blunted by Genotype and Age

**Figure 6:**
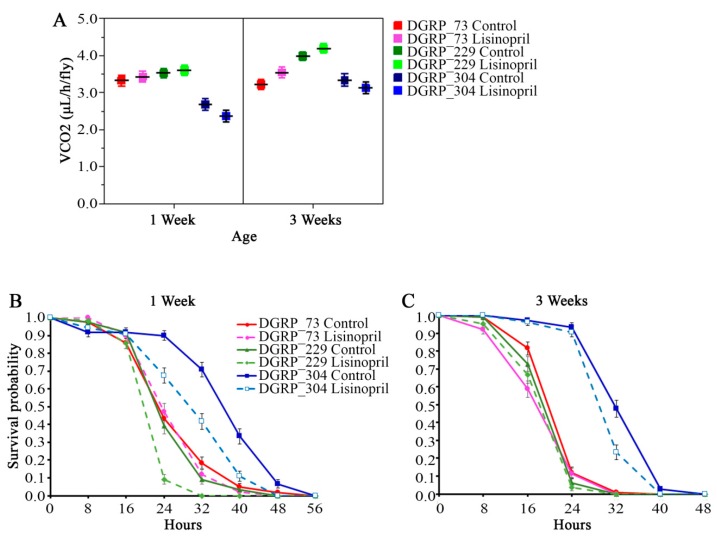
Lisinopril negatively affects survival under nutrient starvation but the effect of the drug can be blunted by genotype and age. (**A**) There is no significant effect of Lisinopril on resting metabolic rate (F_1,106_ = 0.14, *p* = 0.7082). Values represent the least-square means of whole-body CO_2_ production, an index of resting metabolic rate, adjusted for live body weight (*n* = 10 independent replicates). (**B**,**C**) Kaplan–Meier survival probability curves for one-week-old (panel **B**) and three-week-old (panel **C**) DGRP flies fed Lisinopril or control food. There is a significant genotype-by-age-by-Lisinopril interaction effect on survivorship (Wald χ^2^ = 7.53, *p* = 0.0061) in the analysis of pooled data. DGRP_229 and DGRP_334 fed Lisinopril are significantly more sensitive to starvation conditions than control flies at one week of age (Bonferroni corrected log-rank χ^2^ = 28.00, *p* < 0.0001 and χ^2^ = 30.19, *p* < 0.0001, respectively) (panel **B**) but not at three weeks of age (panel **C**). No statistically significant differences were observed for DGRP_73 flies.

Given that Lisinopril induces changes in thoracic mitochondrial and metabolic parameters, we next sought to test whether it impacts nutrition-relevant organismal traits, such as whole-body resting metabolic rate and the fly’s capacity to survive under nutrient starvation. There was no effect of Lisinopril on resting metabolic rate (Figure 6A, Supplementary Table S2). On the other hand, we found that having a specific genotype or age decreased the hazard of death for flies that received the treatment (see Figure 6B,C).

## 3. Discussion

Studies across a broad range of species have established a common set of evolutionarily conserved hallmarks of aging, including an age-related decline in mitochondrial function and increase in ROS production [27]. This evidence points to the potential for pharmacological intervention to improve health span and extend longevity. To this end, strong evidence suggests that pharmacological inhibition of Ang II formation and action is not only beneficial in patients with hypertension, cardiovascular diseases, and diabetic nephropathy but also displays age-retarding effects in humans and models systems [9]. The mechanisms through which blockade of the bioactive component of RAS impacts the aging process and age-related diseases remain largely unknown. However, there is a growing consensus that the beneficial effect of RAS blockade involves a reduction in ROS production and thereby the maintenance of mitochondrial function and content with advancing age [9,10,12]. Here, we took advantage of the evolutionary conservation of ACE across species to study the effects of the ACEi Lisinopril on mitochondrial function and content, H_2_O_2_ levels, and the metabolome in the thorax of the invertebrate model *D. melanogaster* at one-week and three-weeks of age. We reasoned that the use of a model with an open circulatory system might provide important insights into the direct cellular effects of the drug. 

Supporting the mammalian data, we report that Lisinopril treatment reduces *Drosophila* thoracic mitochondrial respiration and H_2_O_2_ levels and enhances mitochondrial content. However, the effects of Lisinopril on these traits are context-dependent and appear only in specific genotypic backgrounds and ages. While the drug effects on mitochondrial respiration and H_2_O_2_ levels are observed in young flies of two different strains (DGRP_73 and DGRP_229, respectively), those on mitochondrial content are found in older flies of another strain (DGRP_304). Accordingly, we also depicted 37 thoracic metabolite features with levels that vary significantly in response to Lisinopril in a genotype- and age-dependent manner. Several of the latter metabolites include phospholipids and long-chain fatty acids, such as 1-palmitoyl lysophosphatidic, Lyso-PE (0:0/18:0), 3-hydroxy-tetradecanoic acid, and hexadecanedioic acid, whose levels are reduced by the AnCe/ACEi drug (see Figure 3). It is well recognized that mitochondria are gatekeepers for cell bioenergetics in most eukaryotic cells [28]. Cellular respiration is regulated by the need for ATP and the balance with other functions of the mitochondria. A pivotal role of mitochondria is in the regulation of cellular lipid homeostasis and disruption of this crosstalk can lead to physiological/pathological changes that are responsible for the aging process and age-related chronic diseases [29]. Mitochondria orchestrate the synthesis of key membrane phospholipids, such as PE, which in turn have many essential biological functions in cells [30]. PE are a class of phospholipids, which together with phosphatidylinositol (PI) and phosphatidylserine (PS) moieties, form the backbone of most biological membranes of both eukaryotic and prokaryotic cells [30]. Mitochondrial PE as well as lysophosphatidic acid, cardiolipin, and the enzymes that generate or catabolize them are involved in the regulation of mitochondrial morphology (e.g., the balance between fusion and fission events) and function [30]. For example, it has been reported that increased PE content induces autophagy and enhances longevity from yeast to mammals [31]. On the other hand, depletion of the mitochondrial content of PE affects mitochondrial fusion, mitochondrial ultrastructure, dynamics, and function [30]. Increases in mitochondrial PE content and/or decreases in the molar ratio of PC/PE positively correlated to ATP content in mammalian hepatocytes and can modulate glucose production [32]. It is, therefore, plausible that Lisinopril-induced changes in the abundance of PE, such as the reduced levels of Lyso-PE (0:0/18:0) in DGRP_73 treated young flies, might in part explain the observed genotype- and age-specific effects of Lisinopril on mitochondrial function and content, most likely through genetic mechanisms that involve changes in mitochondrial structure and function. 

In the present study, we also provide evidence that the genetically based variation in survival under starvation stress in response to Lisinopril treatment might drive the drug-induced changes in mitochondrial function in specific genotypes. Indeed, while young DGRP_229 and DGRP_304 flies fed Lisinopril survived less under starvation conditions compared to control flies, there was no difference between young DGRP_73 untreated and treated flies. Lisinopril-treated DGRP_73 flies also exhibited lower mitochondrial state 3 respiration at one-week of age compared to their age-matched control counterparts, suggesting that the reduction in thoracic mitochondrial OxPhos capacity triggered by the Lisinopril treatment could be a metabolic adaptation that allows the young DGRP-73 flies to survive longer under nutrition stress. However, further work using the entire set of DGRP strains needs to be performed to confirm this speculation.

Another important finding of our study is that Lisinopril perturbs the thoracic metabolic network structure. Among the metabolic networks affected by Lisinopril, we observed enrichment for pathways related to glutaryl-CoA degradation and the mevalonate metabolic pathway. The administration of combined drugs, such as statins and ACEi, is commonly used for the prevention and treatment of cardiovascular diseases due to their vasoprotective role [33]. Studies in animal models suggest that statins and ACEi are strongly connected through the regulation of the mevalonate pathway, which is involved in the synthesis of cholesterol and is the best-known target of statins [34]. *Drosophila* does not produce endogenous cholesterol, but statin treatment has been reported to increase the fly lifespan and improve cardiac health [35]. The identification of the mevalonate pathway as one of the metabolic pathways perturbed by Lisinopril in our study not only corroborates its mechanistic role in some of the additive effects of statins and ACEi but also lays emphasis on other functions of the pathway, such as its role in the regulation of mitochondrial function [36].

Other metabolic pathways perturbed by Lisinopril are involved in glycogen degradation and glucose and glucose-1 phosphatase degradation, a finding that echoes studies in rodents showing that ARBs ameliorate skeletal muscle insulin sensitivity [8]. In this regard, one important point that needs to be raised is that although AnCE is evolutionary conserved, *Drosophila* does not have homologs of any other RAS components. Yet, findings in our study argue for the potential existence of a fly equivalent of the vertebrate Ang II/AT_1_ receptor system that is linked to glucose and glycogen metabolism and mitochondrial biology. This idea is strongly supported by previous work showing that administration of the ARB Losartan improved mitochondrial morphology in indirect flight muscles of *Drosophila* mutants of Multiplexin, the only orthologue of vertebrate collagen types XV and XVIII [18]. Collagen types XV and XVIII are proteoglycans present in the extracellular matrix (ECM) that bear glycosaminoglycan chains [18]. An intermediate for the synthesis of glycosaminoglycan chains is d-glucuronic acid. d-glucuronic acid originates from UDP-glucuronic acid (http://www.hmdb.ca/metabolites/HMDB0000935), which is made from UDP-glucose, a precursor also for glycogen synthesis. We found that Lisinopril treatment increased the abundance of d-glucuronic acid in the thorax of DGRP_229 flies as well as levels of AMP (Supplementary Figure S2). These results are intriguing because regulation of glycogen metabolism is crucial in mammalian muscle energetics [37], and AMP is required not only for activation of glycolytic enzymes but also of glycogen phosphorylase through its AMP-binding domain [38]. As such, AMP promotes glycolysis and glycogenolysis, which in turn leads to the production of glucose 1-phosphate and its activation to form UDP-glucose and ultimately d-glucuronic acid. Formation of the muscle-tendon interactions, in vertebrates and invertebrates, creates mechanical forces needed for the maturation of the myotendinous junction and differentiation of the tissue [39]. This ECM remodeling of the junction is critical to protect against the load generated by muscle contraction [39] and an overlap between mechanisms regulating ECM remodeling and the breakdown of glycogen storage would, therefore, make biological sense. Our hypothesis is also supported by the significant increase in the levels of glutamine in the thorax of DGRP_229 treated flies compared to control flies. In humans, glutamine levels increase in skeletal muscle after exercise and the increased glutamine’s availability leads to muscle glycogen accumulation [40]. It is, therefore, possible that Lisinopril might act through the same mechanisms triggered by exercise to increase glutamine and therefore regulate glycogen levels. Given the extensive evidence that RAS blockade improves exercise capacity in elderly people [41], future studies addressing the hypothesis that the Ang II/AT_1_ receptor system might control mitochondrial biology, ECM remodeling, and glycogen metabolism in skeletal muscle are warranted. 

In conclusion, our results provide novel and important insights into the role of ACEi in cellular energy metabolism and establish *D. melanogaster* as a valuable model to better elucidate underlying mechanisms involved in the beneficial effects of these drugs on the aging process and age-related decline in physiological functions.

## 4. Materials and Methods

### 4.1. D. melanogaster Strains and Rearing Conditions

We obtained the three wild-derived inbred DGRP strains, DGRP_73, DGRP_229, and DGRP_304, from the laboratory of Jeff Leips at UMBC. We reared flies in vials containing 10 mL of standard cornmeal, agar, molasses, and yeast medium, at a constant temperature of 25 °C, 60–75% relative humidity, and 12/12 h light/dark cycle. To perform the experiments described below, male virgin flies were either fed a standard medium (Control groups) or received 1 mM Lisinopril (Sandoz Pharmaceuticals. Princeton, NJ, USA) through its addition to the standard medium for one-week or three-weeks. The 1 mM concentration is equivalent to the dose previously used by Momota and colleagues [18] to show a Losartan effect on muscle mitochondrial morphology. 

### 4.2. Lisinopril Measurement Assay

We confirmed drug uptake in all three DGRP strains through quantification of Lisinopril in whole flies using liquid chromatography–tandem mass spectrometry (LC-MS/MS) (see Supplementary Figure S1). We homogenized thoraces with 5 mM ammonium acetate buffer. Calibration standards, blanks, and Quality Controls (QCs) were prepared by spiking naïve homogenate (100 µL) with the appropriate amount of Lisinopril to achieve concentrations in the tissue homogenate ranging from 50–10,000 ng/mL. Standards, blanks, QCs, and experimental samples were spiked with an internal standard (10 µL of a 100 ng/mL Enalaprat), and proteins were precipitated by the addition of 0.5 mL of 90:10 methanol:acetone solution. After centrifugation for 5 min at 21,000× *g*, the supernatant was transferred to culture tubes and evaporated under a stream of dry nitrogen at 50 °C. The residue was dissolved in DI water, vortexed, transferred to a limited volume autosampler vial, and analyzed in positive ion mode by LC–MS/MS. Detection was performed using an Applied BioSystems 4000 QTRAP (Applied Biosystems, Foster City, CA, USA) triple quadrupole mass spectrometer. Mass calibration, data acquisition, and data quantitation were performed using Applied Biosystem Analyst 1.6.2 software (Applied Biosystems, Foster City, CA, USA).

### 4.3. Mitochondrial Function Assay

We performed mitochondria isolation and respiration assays as previously described in [19], with some modifications. Briefly, respiration rates were determined at 25 °C in respiration buffer (120 mM KCl, 5 mM KH_2_PO_4_, 3 mM Hepes, 1 mM MgCl_2_, and 0.2% BSA, pH 7.2) supplemented with 1 mM EGTA, using Oroboros Oxygraph-2k (O2k, OROBOROS Instruments, Innsbruck, Austria) with pyruvate 5 mM/proline 5 mM as complex I respiratory substrates. State 2 respiration was measured after addition of 1.3 mg of mitochondria and complex I substrates; state 3 respiration was induced by adding ADP (100 M), and state 4 respiration was measured after adding oligomycin 16 g/mL to inhibit ATP synthase. Mitochondrial loading was determined from protein content measured using the BioRad DC assay (Hercules, CA, USA). Citrate synthase activity was measured as described in [19].

### 4.4. Mitochondrial DNA (mtDNA)/Nuclear DNA (nDNA) Ratio Assay

We isolated total DNA from 10 pooled thoraces using NaOH at 95 °C for 30 min followed by neutralization with Tris-HCl. Quantitative PCR was performed in triplicate using SYBR Green Master mix (Bio-Rad), primers for mitochondrial *16S rRNA* (F-AAAAAGATTGCGACCTCGAT; R-AAACCAACCTGGCTTACACC) and nuclear *RpL32* (F-AGGCCCAAGATCGTGAAGAA; R-TGTGCACCAGGAACTTCTTGAA) genes, on a 384 iCycler (Bio-Rad). We calculated the mtDNA/nDNA ratio by the comparative threshold method [42].

### 4.5. H_2_O_2_ Measurement Assay

We dissected five thoraces per genotype, age, and treatment between 10:00 a.m. and 11:00 a.m. from live flies in freshly prepared 20 mM *N*-ethylmaleimide. Thoracic H_2_O_2_ levels were quantified using the Fluorimetric Hydrogen Peroxide Assay Kit (Sigma-Aldrich#MAK165-1KT, St. Louis, MO, USA) according to the manufacturer’s instructions. Fluorescence (λ_ex_ = 540/λ_em_ = 590 nm) was measured with a BioTek microplate reader (BioTek Instruments, Winooski, VT, USA).

### 4.6. Resting Metabolic Rate

We measured metabolic rate as CO_2_ production using a flow-through respirometry system (Qubit System Research, Kingston, ON, Canada) and the protocol described in [43]. 

### 4.7. Starvation Survival Assay

We placed four groups of 10 flies per genotype, age, and treatment on 1.5% agarose medium and the number of flies alive was recorded at 8-h intervals until they were all dead. Three independent sets of starvation survival experiments were performed.

### 4.8. Statistical Analysis

We used a general linear model implemented in SAS (PROC GLM, SAS V9.4) to analyze our data and investigate the main effects of Lisinopril treatment, genotype, age, and all possible interaction terms on mitochondrial respiration rates, mtDNA/nDNA, and H_2_O_2_ levels. The covariate live body weight was included in the model used to analyze resting metabolic rate. A log^10^ transformation was applied to state 2 respiration, state 4o respiration, and H_2_O_2_ data to meet the assumption of normality before the model was run. The Tukey test for post hoc pairwise comparisons was also run to assess significant differences between groups. 

We used Cox regression models as implemented by SAS (PROC PHREG, SAS V9.4) to analyze survivorship data, with genotype, age, Lisinopril, replicate groups, independent experiments, and their interaction terms used as covariates. 

### 4.9. Global Metabolomics Profiling

#### 4.9.1. Metabolite Detection

We performed high-resolution LC–MS analysis for global metabolite profiling. Samples consisted of 36 thoraces (three genotypes, two ages, two treatments, and three replicates in each treatment/genotype/age group), which were flash frozen in liquid nitrogen between 10:00 a.m. and 11:00 a.m. in the De Luca lab, and then sent to the Northwest Metabolomics Research Center in Seattle, WA. Samples were thawed at room temperature, and the protein was precipitated using a cold methanol–water extraction, following previously described methods [44]. 

Each *Drosophila* sample was weighed and then homogenized in 200 µL water with 10% PBS (1×) in an Eppendorf tube while immersed in an ice bath. Methanol (800 µL) was then added, followed by vortexing for 2 min to precipitate proteins and incubation at −20 °C for 30 min. Samples were sonicated in an ice bath for 10 min and then centrifuged at 14,000 rpm for 5 min at 4 °C. From each tube, 900 µL supernatant was transferred to a new Eppendorf tube for drying under vacuum at 30 °C (~3 h). The completely dried samples were reconstituted in 100 μL 40% water/60% ACN for MS analysis. A pooled QC sample was then made by combining small aliquots (~5 µL) from each reconstituted sample. This pooled QC was analyzed once for every 10 study samples to serve as a technical replicate throughout the data set to assess process reproducibility and allow for data normalization to account for any instrument drift. LC-MS analysis was performed using an LC-QTOF-MS system (Agilent Technologies, Santa Clara, CA, USA) consisting of an Agilent 1200 SL liquid chromatography system coupled online with an Agilent 6520 time-of-flight mass spectrometer. A 5 μL aliquot of the reconstituted sample was injected onto a 2.1 × 150 mm Waters BEH-Amide 2.5 μm particle column at 35 °C. The metabolites were gradient-eluted at 0.3 mL/min using mobile phase A, 5 mM ammonium formate and 0.0125% formic acid in 97% water/3% ACN, and mobile phase B, 5 mM ammonium formate and 0.0125% formic acid in 3% water/97% ACN (98% B for 1 min, 98 to 77% B in 6.5 min, 77 to 39% B in 4.5 min and 39% B for 7 min). The MS interface capillary was maintained at 325 °C with a nebulizing gas pressure of 45 psig, and a drying gas flow of 9 L/min. The capillary voltage for positive ion injection was 3.5 kV. LC-MS data were processed using Agilent Mass Profiler Professional (version 13.1.1) for compound identification. A list of ion intensities for each detected peak was generated using a retention time index and *m*/*z* data as the identifiers for each ion. Agilent MassHunter Workstation Data Acquisition software B.02.01 (B2116.30) was used to acquire all data from 60 to 1000 *m*/*z* using centroid mode with a threshold of 200 or 0.01%.

#### 4.9.2. Data Analysis

##### Data Pre-Processing

We first performed a median normalization where we adjusted the data so all samples would have the same median value of the metabolite abundance post log^2^ transformation. We then selected metabolites with ≤5% missingness and imputed the remaining missing data using the *K*-nearest neighbor (KNN) algorithm using the Bioconductor impute package [45]. Briefly, for each metabolite with missing values, we found the KNN (where *K* = 10) using a Euclidean distance, confined to the columns (samples) for which that metabolite is not missing. For every metabolite, the missing values were then imputed using the average of the non-missing values of its neighbors.

##### Multivariate and Univariate Analyses

We implemented principal component (PC) analysis (PCA) using the vegan package in R (version 3.5.0). PCA was performed combining both positive and negative metabolic features to determine how much of the thoracic metabolome variance is explained by genotype, age, and treatment. 

To examine main effects of genotype, age, and treatment, and interaction effects on each of the metabolic features from the positive and negative mode, we fitted a weighted linear model to the data using the Bioconductor limma package [46]. The limma package uses empirical Bayes moderated statistics, which improves power by “borrowing strength” between metabolites in order to moderate the residual variance [47]. The sample-specific weights were computed using the array Weights function from the limma package. This allowed us to up or down-weight individual samples. Metabolite changes were considered significant with a false discovery rate (FDR) of 10% to account for multiple testing (e.g., ~90% of the hits that we called are true positives). Since there are three genotypes, we performed a moderated *F*-test either when we tested the genotype as the main effect or when there was more than one interaction term involving genotype in the model.

##### Network Analysis

To identify modules of metabolites differentially co-expressed between Lisinopril and control treatments, we applied the differential co-expression method, DiffCoEx [48], which takes advantage of methods from the Weighted Gene Network Correlation Analysis (WGCNA) package in R [49]. Briefly, WGCNA generates a correlation matrix of all metabolic features across all observations from the dataset and applies a clustering algorithm to identify clusters (or modules) of related features. DiffCoEx takes these identified modules and evaluates the difference in their abundance levels across two environments (in our case, Lisinopril vs. control) to identify modules that are differentially regulated across these two environments. Different input parameters can be adjusted when using DiffCoEx. In our analysis, we set the scaling coefficient *β* = 7 and set the minimum module size to 20 features. DiffCoEx identifies modules of features that show similar changes between treatment and control. We used the software package *Mummichog* [26] to assign pathway Ids and to test for functional enrichment within each set of metabolite features associated with a specific module. 

## Supplementary Materials

The following are available online at http://www.mdpi.com/1422-0067/19/11/3351/s1, Figure S1. Lisinopril concentration in whole-body homogenates, Table S1. Analyses of variance of thoracic mitochondrial function traits in young and middle-aged Lisinopril treated and control flies, Table S2. Analyses of variance and covariance of thoracic mitochondrial content, reactive oxygen species, and whole-body resting metabolic rate in young and middle-aged Lisinopril treated and control flies, Table S3. List of metabolites detected by LC–MS in the thoraces of three Drosophila Genome Reference Panel strains at one and three weeks of age, Table S4. Untargeted metabolomics analysis results, Table S5. *Mummichog* pathway enrichment analysis for modules of differentially co-regulated metabolite features between Lisinopril treated and control flies.
