# Supplementary material for: Age- and Genotype-Specific Effects of the Angiotensin-Converting Enzyme Inhibitor Lisinopril on Mitochondrial and Metabolic Parameters in Drosophila melanogaster
Source: Int J Mol Sci. 2018 Oct 26;19(11):3351. doi: 10.3390/ijms19113351 (PMC6274988; doi:10.3390/ijms19113351)
Supplement: Supplementary file 1 [file ijms-19-03351-s001.zip › Supplementary Material/Supplementary Table 1.docx]

**Supplementary Table 1. Analyses of variance of thoracic mitochondrial function traits in young and middle-aged Lisinopril treated and control flies.** df: degrees of freedom; SS: Type III Sums of Squares, MS: Mean square;

| **Phenotype** | **Source of Variation** | **df** | **SS** | **MS** | **F-value** | ***P*-value** |
| --- | --- | --- | --- | --- | --- | --- |
| **State 3 respiration rate** | Treatment | 1 | 16842.0092 | 16842.0092 | 2.75 | 0.1021 |
|  | Genotype | 2 | 264924.3832 | 132462.1916 | 21.63 | <.0001 |
|  | Age | 1 | 230097.4087 | 230097.4087 | 37.57 | <.0001 |
|  | Treatment*Genotype | 2 | 42649.1340 | 21324.5670 | 3.48 | 0.0366 |
|  | Treatment*Age | 1 | 18008.6176 | 18008.6176 | 2.94 | 0.0912 |
|  | Genotype*Age | 2 | 312995.0536 | 156497.5268 | 25.55 | <.0001 |
|  | Treatment*Genotype*Age | 2 | 43712.2959 | 21856.1479 | 3.57 | 0.0338 |
|  | Error | 65 | 398106.718 | 6124.719 |  |  |
|  |  |  |  |  |  |  |
| **State 2 respiration rate** | Treatment | 1 | 0.1371 | 0.1371 | 1.40 | 0.2404 |
|  | Genotype | 2 | 4.1526 | 2.0763 | 21.25 | <.0001 |
|  | Age | 1 | 3.6535 | 3.6535 | 37.39 | <.0001 |
|  | Treatment*Genotype | 2 | 0.3727 | 0.1863 | 1.91 | 0.1567 |
|  | Treatment*Age | 1 | 0.2902 | 0.2902 | 2.97 | 0.0896 |
|  | Genotype*Age | 2 | 3.7465 | 1.8733 | 19.17 | <.0001 |
|  | Treatment*Genotype*Age | 2 | 0.4737 | 0.2369 | 2.42 | 0.0965 |
|  | Error | 65 | 6.3507 | 0.0977 |  |  |
|  |  |  |  |  |  |  |
| **State 4_o_ respiration rate** | Treatment | 1 | 0.0044 | 0.0044 | 0.02 | 0.6478 |
|  | Genotype | 2 | 4.6204 | 2.3102 | 10.36 | 0.0001 |
|  | Age | 1 | 1.5812 | 1.5812 | 7.09 | 0.0098 |
|  | Treatment*Genotype | 2 | 0.1950 | 0.0975 | 0.44 | 0.6478 |
|  | Treatment*Age | 1 | 0.0727 | 0.0727 | 0.33 | 0.5701 |
|  | Genotype*Age | 2 | 4.7113 | 2.3557 | 10.56 | 0.0001 |
|  | Treatment*Genotype*Age | 2 | 0.4049 | 0.2024 | 0.33 | 0.5701 |
|  | Error | 63 | 14.0488 | 0.2230 |  |  |

State 2 and oligomycin-induced state 4 (state 4_o_) respiration data were log_10_ transformed to fulfill the assumption of normality
