# Supplementary material for: Age- and Genotype-Specific Effects of the Angiotensin-Converting Enzyme Inhibitor Lisinopril on Mitochondrial and Metabolic Parameters in Drosophila melanogaster
Source: Int J Mol Sci. 2018 Oct 26;19(11):3351. doi: 10.3390/ijms19113351 (PMC6274988; doi:10.3390/ijms19113351)
Supplement: Supplementary file 1 [file ijms-19-03351-s001.zip › Supplementary Material/Supplementary Table 2.docx]

**Supplementary Table 2. Analyses of variance and covariance of thoracic mitochondrial content, reactive oxygen species, and whole-body resting metabolic rate in young and middle-aged Lisinopril treated and control flies.** mtDNA/nDNA: mitochondrial DNA/nuclear DNA; H_2_O_2_: hydrogen peroxide; df: degrees of freedom; SS: Type III Sums of Squares, MS: Mean square;

| **Phenotype** | **Source of Variation** | **df** | **SS** | **MS** | **F-value** | ***P*-value** |
| --- | --- | --- | --- | --- | --- | --- |
| **mtDNA/nDNA** | Treatment | 1 | 0.0027 | 0.0027 | 1.06 | 0.3070 |
|  | Genotype | 2 | 0.0029 | 0.0014 | 0.58 | 0.5637 |
|  | Age | 1 | 0.0279 | 0.0279 | 11.17 | 0.0015 |
|  | Treatment*Genotype | 2 | 0.0098 | 0.0049 | 1.96 | 0.1506 |
|  | Treatment*Age | 1 | 0.0128 | 0.0128 | 5.10 | 0.0282 |
|  | Genotype*Age | 2 | 0.0206 | 0.0103 | 4.12 | 0.0218 |
|  | Treatment*Genotype*Age | 2 | 0.0221 | 0.0110 | 4.41 | 0.0170 |
|  | Error | 52 | 0.1300 | 0.0025 |  |  |
|  |  |  |  |  |  |  |
| **H_2_O_2_ levels** | Treatment | 1 | 1.004 | 1.004 | 10.30 | 0.0017 |
|  | Genotype | 2 | 2.973 | 1.487 | 15.25 | <.0001 |
|  | Age | 1 | 3.330 | 3.330 | 34.16 | <.0001 |
|  | Treatment*Genotype | 2 | 0.4887 | 0.2443 | 2.51 | 0.0859 |
|  | Treatment*Age | 1 | 0.7337 | 0.7337 | 7.52 | 0.0070 |
|  | Genotype*Age | 2 | 2.0748 | 1.0374 | 10.64 | <.0001 |
|  | Treatment*Genotype*Age | 2 | 0.5803 | 0.2901 | 2.98 | 0.0500 |
|  | Error | 117 | 11.4063 | 0.0975 |  |  |
|  |  |  |  |  |  |  |
| **VCO_2_** | BW | 1 | 6.5669 | 6.5669 | 36.87 | <.0001 |
|  | Treatment | 1 | 0.0251 | 0.0251 | 0.14 | 0.7082 |
|  | Genotype | 2 | 15.5239 | 7.7620 | 43.58 | <.0001 |
|  | Age | 1 | 4.8684 | 4.8684 | 27.34 | <.0001 |
|  | Treatment*Genotype | 2 | 0.8783 | 0.4392 | 1.83 | 0.1653 |
|  | Treatment*Age | 1 | 0.1444 | 0.1444 | 0.81 | 0.3700 |
|  | Genotype*Age | 2 | 2.2424 | 1.1212 | 6.30 | 0.0026 |
|  | Treatment*Genotype*Age | 2 | 0.0192 | 0.0100 | 0.81 | 0.3700 |
|  | Error | 106 | 18.8778 | 0.1781 |  |  |

BW: Live Body Weight measured to 0.01 mg accuracy with an analytical balance was used as a covariate. H_2_O_2_ data were log_10_ transformed to fulfill the assumption of normality
