# Supplementary figures and images for: Age- and Genotype-Specific Effects of the Angiotensin-Converting Enzyme Inhibitor Lisinopril on Mitochondrial and Metabolic Parameters in Drosophila melanogaster
Source: Int J Mol Sci. 2018 Oct 26;19(11):3351. doi: 10.3390/ijms19113351 (PMC6274988; doi:10.3390/ijms19113351)

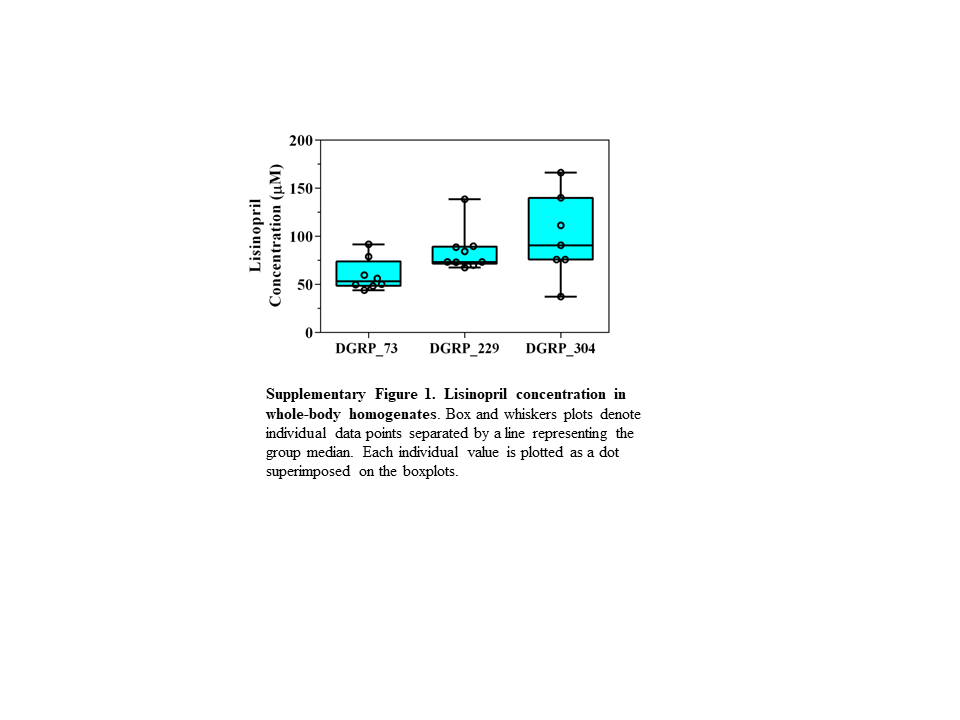

Supplement: Supplementary file 1 [file ijms-19-03351-s001.zip › Supplementary Material/Supplementary Figure 1.tif]
